# Knowledge and associated factors towards diabetes mellitus among adult non-diabetic community members of Gondar city, Ethiopia 2019

**DOI:** 10.1371/journal.pone.0230880

**Published:** 2020-03-26

**Authors:** Abiy Maru Alemayehu, Henok Dagne, Baye Dagnew

**Affiliations:** 1 Department of Optometry, College of Medicine and Health Sciences, University of Gondar, Gondar, Ethiopia; 2 Department of Environmental and Occupational Health and Safety, Institute of Public Health, College of Medicine and Health Sciences, University of Gondar, Gondar, Ethiopia; 3 Department of Human Physiology, College of Medicine and Health Sciences, University of Gondar, Gondar, Ethiopia; Boston University, UNITED STATES

## Abstract

**Introduction:**

Diabetes mellitus is a metabolic disorder resulting from either loss of insulin producing cells, insufficient insulin action, or both. Knowledge can play an important role in preventing diabetes mellitus and its complications. There is limited information regarding knowledge and related factors regarding diabetes mellitus among non-diabetic adult community members in the study area. Therefore, the current study aimed to determine knowledge and associated factors towards diabetes mellitus among non-diabetes community members of Gondar city.

**Methods:**

Community-based cross-sectional study was conducted on non-diabetic community members from July 1–29, 2019 in Gondar city. The participants were selected randomly from each households. A structured questionnaire was used to collect the data. EpiData version 3.1 was used for data entry and SPSS version 20 was used for data processing and analysis. Descriptive statistics were calculated for most variables. Multivariable logistic regression was used to identify the associated factors. A variable was considered significantly associated at p-value<0.05.

**Result:**

A total of 633 study subjects participated in this study with a mean age of 36.12 (± 12.87) years. Of these study participants, 572 had awareness about diabetes mellitus and 51.4% (95% CI: 47.4%, 55.8%) had good knowledge. Being male [Adjusted odds ratio = 1.62 (95% CI: 1.05, 2.48)], monthly income of 3000–5000 birr [Adjusted odds ratio = 1.88 (95% CI: 1.03, 3.41)], monthly income of ≥5001 birr [Adjusted odds ratio = 2.37 (95% CI: 1.17, 4.78)], previous training on diabetes mellitus [Adjusted odds ratio = 4.37 (95% CI; 3.04, 7.37)], being grade 9–12 [Adjusted odds ratio = 3.1 (95% CI: 1.09, 8.66)], having college and above educational qualification [Adjusted odds ratio = 3.70 (95% CI: 1.26, 10.85)] were significantly associated with good knowledge towards diabetes mellitus.

**Conclusion:**

The level of knowledge regarding diabetes mellitus was low among study participants which indicates a need for health education intervention. Previous training on diabetes mellitus, educational status and average monthly income and being male were the factors associated with good knowledge of participants about diabetes mellitus.

## Introduction

Diabetes mellitus (DM) is a metabolic disorder which is characterized by increased blood glucose level with disturbances of carbohydrate, fat and protein metabolism resulting from either loss of insulin producing cells, insufficient insulin action, or both [1]. World health organization (WHO) projects the number of patients with diabetes mellitus to be 366 million by the year 2030 which is more in low- and middle-income countries [2]. However, the International Diabetes Federation estimates this number to be 472 million by 2030 which indicates how the prevalence increases from time to time[3]. It is one of the public health threats in the world. Diabetes is an important public health problem, one of four priority non-communicable diseases targeted for action by world leaders, that leads to visual impairment, heart attack, stroke, kidney dysfunction, amputation, and nerve damage [4]. Diabetes leads to blinding ocular problems if it is left undiagnosed in the early stage and not treated accordingly [5]. In addition to its physical complications among individuals, DM has several negative impact on the nation’s economy [4].

Most societies lack awareness about DM and its tremendous complications [5]. Creating awareness can enabling a population to have a better understanding of DM. Thus, it can help in reducing the complications, unwanted impact of the condition and health care costs due to DM. Awareness creation programs about DM have always helped to prevent and manage DM [6,7].

Knowledge about DM can play an important role to encourage the community for the prevention and minimization of complications due to DM [7,8].

A great variation exists in knowledge towards DM among community members, but the reasons are not clear. However the level of knowledge towards DM has been associated with socio-demography, educational status [9], family history of DM [10], previous training on DM, source of information and other factors.

Although it is a common public health problem, there was no sufficient data about knowledge and associated factors towards DM in the study area among non-diabetic community members. Therefore, this study aimed to determine knowledge and factors towards diabetes mellitus among non-diabetic individuals.

## Methods and materials

### Study design, area and period

A community-based cross-sectional study was conducted in Gondar city, Northwest Ethiopia, from July 1–29, 2019. Information obtained from Central Gondar zone administration finance and economic office indicated that, Gondar city is located in the Central Gondar zone which is situated 748 km from the capital city, Addis Ababa. According to the office, it had a population of 351, 675 divided into 10 sub-cities and 24 kebeles (smallest administrative units in Ethiopia) [11]. There was one government hospital-that is University of Gondar comprehensive specialized hospital.

### Source and study population

All adult non-diabetic community members living in Gondar city who were present at households during the data collection period.

### Inclusion and exclusion criteria

#### Inclusion criteria

All adult non-diabetic community members in Gondar city who were found at home during the data collection period

#### Exclusion criteria

Diabetic patients and patients with other chronic systemic illness

Sample size determination

The sample size was calculated by taking variability of proportion from a similar study which was 52.5% for knowledge [12]. Taking 351, 675 community members as source population and using Open Epi software package for the determination of sample size, which uses the formula given below for the finite population [13], the final sample size was calculated as:

$$n=\frac{Nz^{2}pq}{d^{2}(N-1)+z^{2}pq}$$

Where n = sample size

N = source population

P = proportion of knowledgeable non-diabetic community

d = margin of error

z = Value of z statistic at 95% confidence interval = 1.96

The sample size became 383.

After considering 10% non-response rate for any unpredictable events and design effect of 1.5, the final required sample size was 633.

#### Sampling technique and procedures

To ensure representativeness, the sample was taken from about 25% of the total 24 kebeles. Six kebeles were selected using the lottery method. In the selected 6 kebeles there were a total of 16, 396 households. A list of each household was taken from each Kebele administration. The study participants were selected using a systematic random sampling technique by calculating the intervals K using the formula K = N/n, i.e.16, 396 /633 = 26

Where, n = sample size, N = total number of households as listed from each kebeles. Consequently, by selecting one house out of 26 households using the lottery method as a starting point every 26^th^ house was used to conduct the study directly. If the selected household is inaccessible, the next household was included. When more than one study participants found in the selected household, the lottery method was used to select one participant. The interview was conducted after receiving permission from the household heads.

**Fig 1:**
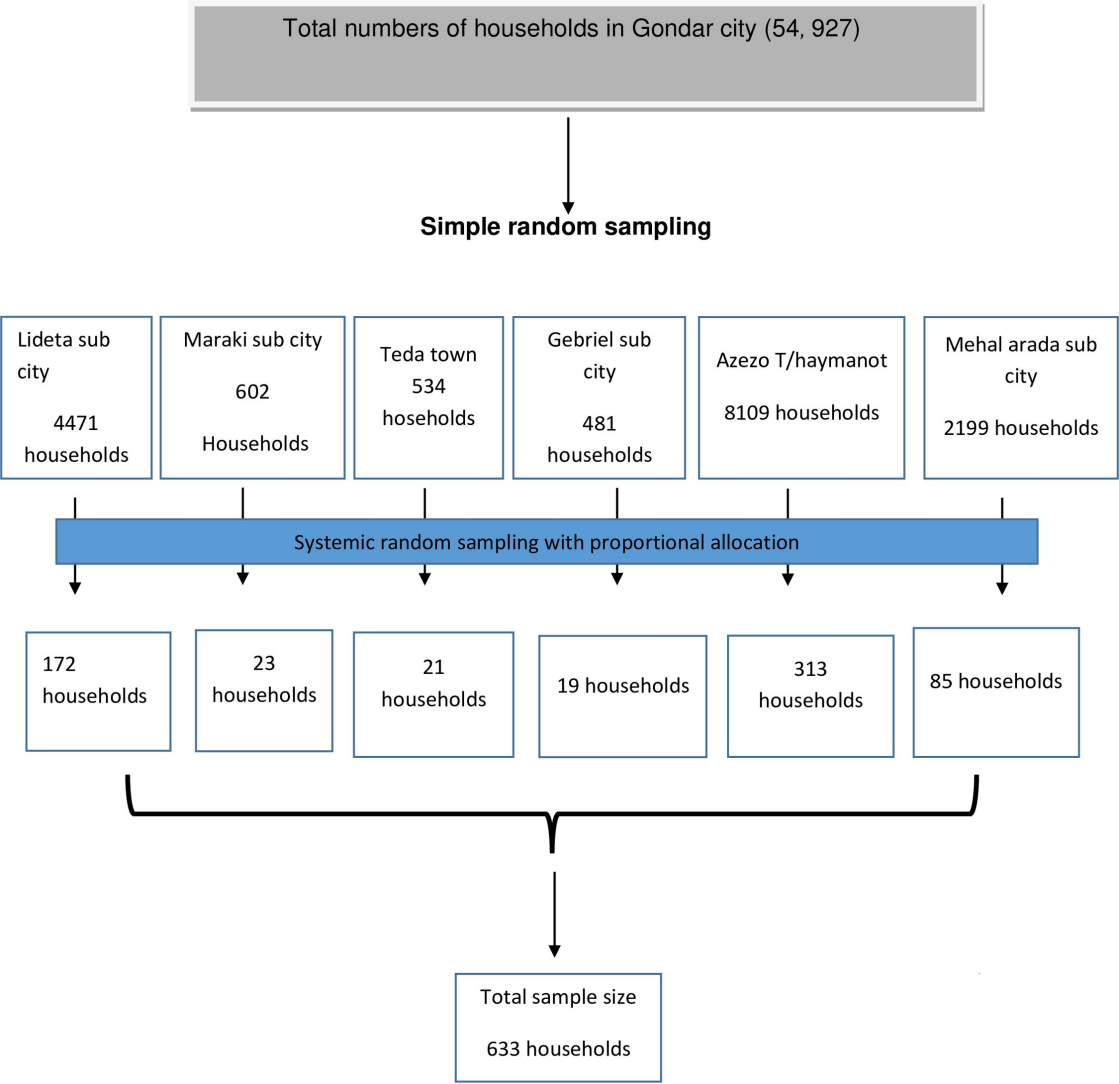
Schematic representation of the sampling procedure and technique.

Fig 1. The diagrammatic representation of the sampling procedure and technique.

The study was conducted after ethical clearance was obtained from the ethical review board of the University of Gondar with the reference number of V/P/RCS/05/2030/3019. Officials at the school of Medicine and Gondar city administration were communicated through formal letters that was obtained from the ethical review board of the University of Gondar. Written informed consent was obtained from each study participants. Participants were informed about the objective of the study and their full right to discontinue or refuse to participate in the study. The confidentiality of the information obtained was assured through anonymity.

### Operational definition

**Good knowledge**: Individuals who responded the mean (19.37) and above of the total knowledge questions had good knowledge about diabetes mellitus.

**Poor knowledge**: Individuals who responded below the mean (19.37) of the total knowledge questions had poor knowledge about diabetes mellitus.

**Awareness:** A participants were classified as being aware of diabetes mellitus if a positive response (‘Yes’) is obtained to the question ‘have you ever heard of diabetes mellitus?’

**Non-diabetic community member:** A portion of a community member who had no known history of diabetes mellitus.

**Adult:** A person older than 18 years of age.

**Household:** A person or group of related or unrelated persons who lived together in the same dwelling unit(s), who acknowledge one adult male or female as the head of the household, who shared the same housekeeping arrangements, and who were considered a single unit [14].

### Data collection procedures and personnel

A pre-tested structured interviewer administered questionnaire was used after reviewing different works of literature [10,12,15–17]. The questionnaire covered demographic information and general knowledge about diabetes. The reliability of the questionnaire was checked by conducting a pretest in Bahirdar city, which had similar characteristics with Gondar city, by taking 5% of the sample size. From the pretest, understandability, clarity, and organization of the questionnaire were checked. From the reliability test of knowledge questions, 0.868 Cronbach's alpha value was found. The questionnaire with 31 point scale items was prepared in English language and then translated to Amharic (local language in Gondar city) that was used for data collection and re-translated back to English to check its consistencies. The questionnaire was then refined accordingly for final use. The interview was conducted on selected households. Eight trained optometrists have participated in data collection.

The knowledge of study participants about diabetes mellitus was assessed using 31 point scale items. There were 31 multiple choice questions that carried a total of 31 correct responses. Each correct response was given a score of 1 and a wrong response a score of 0. Total points to be scored were 31 and the minimum score was 0. Previous studies were used for the classification of study participants' knowledge level. With a score of mean and above meant a good knowledge and poor knowledge for a score less than mean [10,12].

### Data quality control

Data were collected after training was given for data collectors on how to interview participants. On the field-work, the supervisor closely followed the day-to-day data collection process and ensure the completeness of the collected data. Finally, 5% of the collected samples were checked by the principal investigator daily.

### Data processing and analysis

After cleaning and coding, the data were entered into EPIData version 3.1 and exported to and analyzed using SPSS version 20. Proportions, rates and summary statistics such as mean, the standard deviation were calculated for most variables. Multivariable logistic regression was used to determine the factors associated with knowledge towards diabetes mellitus by entering all independent variables in to the model. The variables with a p-value of less than 0.05 were considered statically significant associated with the outcome.

## Results

### Socio-demographic characteristics of the study participants

A total of 633 study participants took part in the study. About 9.6% were not aware of diabetes mellitus. Therefore, the analysis was done for the remaining 90.4% of participants who were aware of diabetes mellitus. Among them, 52.6% (301) were females. The mean age of the study participants was 35.49 years (± 12.36 years). Close to half of the study participants (48.3%) had college and above educational status (Table 1).

**Table 1:** Socio-demographic characteristics and source of information of study participants in Gondar city, Northwest Ethiopia, 2019 (n = 633).

Variables	Frequency	Percent
Have heard of diabetes mellitus		
Yes	572	90.4
No	61	9.6
Sex		
Female	271	47.4
Male	301	52.6
Age category in years		
≤ 24	101	17.7
25–30	153	26.7
31–40	159	27.8
≥41	159	27.8
Educational status		
Cannot read and write	33	5.8
Can read and write	64	11.2
Grades 1–8	34	5.9
Grades 9–12	165	28.8
College and above	276	48.3
Occupational status		
House wife	86	15.1
Student	98	17.1
Merchant	120	21.0
Farmer	17	3.0
Civil servant	214	37.4
Daily laborer	37	6.5
Monthly income in ETB		
≤ 1999	86	15.1
2000–2999	145	25.3
3000–5000	235	41.1
≥ 5001	106	18.5
Previous training on DM		
Yes	178	31.1
No	394	68.9
Family history of DM		
Yes	79	138
No	493	86.2

### Knowledge towards diabetes mellitus

Out of 572 study participants, 294 (51.4%) (95% CI: 47.4, 55.8) had good knowledge. Most study participants defined diabetes as the presence of high blood sugar levels in the body (78.7%), but nearly half of the study participants did not know DM as a condition of insufficient insulin production. Study participants mentioned being obese (70.5%) as the major risk factor for the development of diabetes. Polyphagia (88.5%), feeling of tiredness (78.1%), and the presence of high blood sugar levels (78.3%) were reported as symptoms of DM (Table 2).

**Table 2:** Frequency distribution of participants' responses of knowledge towards diabetes mellitus, Gondar city, 2019 (n = 572).

Variables	Yes		No		I do not know	
	Nº.	%	Nº.	%	Nº.	%
**What is DM?**						
DM is a condition of insufficient insulin production	261	45.6	31	5.4	280	49.0
DM is a condition of the body which not responding for insulin	289	47.0	32	5.6	271	47.4
DM is a condition of high level of sugar in the blood	450	78.7	21	3.7	101	17.6
DM is not curable	275	48.1	185	32.1	112	19.6
DM is diseases which affect any part of the body	257	44.9	159	27.8	156	27.3
**What are the risk factors of DM?**						
Older age	378	66.3	92	16.1	101	17.6
Genetic or family history of diabetes mellitus	303	53.0	92	16.1	177	30.9
Being Obese	403	70.5	71	12.4	98	17.1
Pregnancy	188	32.9	108	18.9	276	48.2
Poor dietary habits	390	68.2	87	15.2	95	16.6
Not getting enough exercise	447	78.2	46	8.0	79	13.8
**What are the signs and symptoms of DM?**						
Frequent urination	364	63.6	47	8.2	161	28.1
Excessive thirst	373	65.2	52	9.1	147	25.7
Excessive hunger	506	88.5	15	2.6	51	8.9
Weight loss	329	57.5	96	16.8	147	25.7
High blood sugar level	448	78.5	35	6.1	89	15.6
Blurring of vision	302	52.8	48	8.4	222	38.8
Slow healing of cuts and wounds	371	64.9	61	10.7	140	24.5
Feeling of tiredness	447	78.1	34	5.9	91	15.9
**Control and management of DM**						
Insulin injection is available for control and management of DM	502	87.8	11	1.9	59	10.3
Tablets & capsule are available for control and management of DM	377	65.9	35	6.1	160	28.0
Regular Exercise	456	79.7	31	5.4	85	14.9
Practicing healthy diet	484	84.6	42	7.3	46	8.0
Medical eye checkup and care	362	63.5	31	5.4	179	31.3
Feet and toes medical checkup and care	343	60	42	7.3	187	32.7
Weight reduction	382	66.8	44	7.7	146	25.5
**Complications of DM**						
Diabetes can cause eye problem or even blindness	355	62.1	36	6.3	181	31.6
Diabetes can cause kidney failure	251	43.9	53	9.2	268	46.9
Diabetes can cause heart failure	215	37.6	59	10.3	298	52.1
Diabetes can cause brain disease like stroke	178	31.1	62	10.8	332	58.0
Diabetes can result in amputation of limb	415	72.6	32	5.6	125	21.9

N°. Number

### Factors associated with the proportion of knowledge towards diabetes mellitus

Sex, previous training about diabetes mellitus, age, monthly income, educational status, occupational status and presence of a person with diabetes mellitus in the family were entered into the multivariable analysis. Sex being male, an income of 3000–5000 Ethiopian Birr (ETB), 5001 and above, previous history of training on diabetes mellitus and educational status of grades 9–12 and college and above were associated significantly with knowledge (Table 3).

**Table 3:** Factors associated with knowledge of study participants regarding diabetes mellitus in Gondar city, Northwest Ethiopia, 2019 (n = 572).

Variables	Knowledge		AOR (95% CI)	P-value
	Good	Poor		
Sex				
Female	137	164	1.00	
Male	157	114	1.62 (1.053, 2.48)	0.028
Age in years				
≤ 24	51	50	1.53 (0.89, 2.62)	0.123
25–30	79	74	1.01 (0.57, 1.81)	0.967
31–40	93	66	0.75 (0.27, 1.22)	0.149
≥41	71	88	1.00	
Monthly income in ETB				
≤ 1999	31	55	1.00	
2000–2999	61	84	1.56 (0.61, 2.19)	0.652
3000–5000	136	99	1.88 (1.03, 3.41)	0.038
≥5001	66	40	2.37 (1.17, 4.78)	0.016
Educational status				
Can’t read and write	9	24	1.00	
Can read and write	16	48	0.64 (0.22, 1.86)	0.410
Grade 1–8	15	19	2.03 (0.62, 6.65)	0.242
Grade 9–12	89	76	3.10 (1.09, 8.66)	0.033
College and above	165	111	3.70 (1.26, 10.85)	0.017
Occupational status				
House wife	32	54	1.00	
Student	61	37	1.30 (0.53, 3.18)	0.561
Merchant	58	62	0.58 (0.28, 1.19)	0.139
civil servant	121	93	0.69 (0.33, 1.46)	0.334
Daily laborer	22	32	0.81 (0.34, 1.97)	0.653
Previous training about DM				
No	164	230	1.00	
Yes	130	48	4.74 (3.04, 7.37)	0.000
Family history of DM				
No	240	253	1.00	
Yes	54	25	1.77 (0.99, 3.13)	0.052

AOR = Adjusted Odds ratio, 1.00 = Reference

Table 3 shows that the odds of good knowledge regarding diabetes mellitus among male study participants were 1.62 times greater than the odds of good knowledge for women [AOR = 1.62 (95% CI: 1.05, 2.48)].

The odds of good knowledge regarding diabetes mellitus among study participants who had an income of 3000–5000 ETB were 1.88 times greater than the odds of good knowledge among subjects with an income of ≤ 1999 ETB [AOR = 1.88 (95% CI: 1.03, 3.41)]. The odds of good knowledge regarding diabetes mellitus among study participants who had an income of ≥5001 ETB were 2.37 times greater than the odds of good knowledge for those subjects with the income of ≤ 1999 ETB [AOR = 2.37 (95% CI: 1.17, 4.78)].

The odds of good knowledge regarding diabetes mellitus among study participants who had previous training on diabetes mellitus were 4.74 times greater than the odds of good knowledge for study subjects with no history of training on diabetes mellitus [AOR = 4.37 (95% CI; 3.04, 7.37)]. The odds of good knowledge regarding diabetes mellitus among subjects who were grades 9–12 were three times greater than the odds of knowledge for subjects who could not read and write [AOR = 3.1 (95% CI: 1.09, 8.66)]. Similarly, the odds of good knowledge regarding diabetes mellitus among subjects who had college and above educational qualifications were four times greater than the odds of knowledge for subjects who could not read and write [AOR = 3.70 (95% CI: 1.26, 10.85)].

## Discussion

This community based cross-sectional study was conducted to determine the knowledge and identify associated factors of the non-diabetic adult community members regarding diabetes mellitus in Gondar city. In this study 294 (51.4%) (95% CI: 47.4, 55.8) non-diabetic adult community members had good knowledge of diabetes mellitus. This finding was in line with studies conducted in Bale zone administrative town (52.5%), Debretabor town (49%) and India (49.9%) [10,12,18]. The possible reason might be due to the similarity of the study design used, which was a community-based study. But it was lower than the study conducted in Kemissie and Kombolcha towns, Ethiopia [19]. This might be due to the difference in the study population in which the study conducted in kemissie and Kombolcha assess the knowledge levels separately among subjects with and without diabetes family members. In addition to this, it was lower than knowledge level reported in the studies done in India [20], Sri Lanka [21] and Saudi Arabia [9]. This could be due to measurement differences. The study conducted in Saudi Arabia measured the level of knowledge using awareness. On the other hand this result is higher than the study conducted in Kenya(27.2%) [22], India (9.2%) [23] and Sudan(15%) [24]. This discrepancy might be due to the sociocultural difference between these populations. The other possible explanation might be the studies conducted in Kenya, India and Sudan, respondents were from rural setup who may lack adequate information regarding diabetes mellitus.

In this study, a significant number of study participants (9.6%) were not aware of diabetes mellitus. Lack of awareness about diabetes mellitus is common in the poor and illiterate segment of the population as indicated by the study done in Pakistan [17]. The lack of awareness and the knowledge gap existed in the community may increase the burden of the condition. This also indicates the need to advocate on diabetes mellitus to promote public health.

The odds of good knowledge regarding diabetes mellitus among study participants who had previous training on diabetes mellitus were five times greater than the odds of good knowledge for study subjects with no history of training on diabetes mellitus. This finding is agreed with another study done in Bale [12]. This may be due to the fact that training is one of the methods to boost knowledge towards diabetes mellitus as indicated in the research done in Indonesia [7].

The odds of good knowledge regarding diabetes mellitus among study participants who had an income of 3000–5000 ETB were two times greater than the odds of good knowledge for study subjects who had an income of ≤ 1999 ETB. In addition to this, the odds of good knowledge regarding diabetes mellitus among study participants who had an income of 5001 and above ETB were two times greater than the odds of good knowledge for study subjects who had an income of ≤ 1999 ETB. This finding is in line with the studies done in Mekelle and Debre Tabor, Ethiopia [8,10], Bangladesh [25] India [18], and Pakistan [17]. Upper socioeconomic status may increase the exposure of individuals to information about diabetes mellitus. That is why a high level of income has a positive association with knowledge towards diabetes mellitus.

The odds of good knowledge regarding diabetes mellitus among study participants who had the educational status of grades 9–12 were three times greater than the odds of good knowledge for study subjects who could not read and write. And also, the odds of good knowledge regarding diabetes mellitus among study participants who had educational qualifications of college and above were four times greater than the odds of good knowledge for study subjects who could not read and write. This study is comparable with the studies done in Ethiopia [8,19], Sri Lanka [21], Bangladesh [25,26] and Pakistan [17,27]. This may be because of improvement of acquisition of knowledge as the level of education increases.

The odds of good knowledge regarding diabetes mellitus among male study participants was 1.6 times greater than the odds of good knowledge among females. This is in line with the studies done in Bangladesh [26] and Pakistan [17]. Better knowledge about DM was also reported among males in Karachi [15]. The possible explanation may be due to the socio-cultural influence in the community, which indicates the exposure of males for information than females in the study area. Since males are mainly engaged in outdoor activities, the likelihood of exposure to health related information might be higher compared to females. Whereas females are mainly involved in indoor activities [28]. Data from the 2011 Ethiopia demographic and health survey also indicates that 47% of males were exposed to information compared to only 32% females [29]. Significantly higher knowledge scores among males than females may also be related to a higher level of education among males [17]. In this study the illiteracy rate of males was 1.22% as compared to females which was 4.55%. This may again explain why males are more knowledgeable than females towards diabetes mellitus. However in a study conducted in the Bale zone, Ethiopia, sex was not significantly associated with knowledge towards diabetes mellitus [12].

### Limitation of the study

Like most other health studies data from cross sectional studies, by its nature had a defect to detect causes and effect relationships of conditions. Since the study unit was household base, homeless and street individuals were excluded which could affect its generalizability.

## Conclusion

This study showed that only about half the non-diabetic community in Gondar city had good knowledge regarding diabetes mellitus.

Previous training on diabetes mellitus, educational status and monthly family income and being male were the factors associated with good knowledge of participants about diabetes mellitus.

## Supporting information

S1 File Questionnaire and data extraction form to determine knowledge and associated factors towards diabetes mellitus among adult non-diabetes community members of Gondar city, Ethiopia 2019. (DOCX)

S1 Data Original data set. (SAV)
