# Supplementary material for: Knowledge and associated factors towards diabetes mellitus among adult non-diabetic community members of Gondar city, Ethiopia 2019
Source: PLoS One. 2020 Mar 26;15(3):e0230880. doi: 10.1371/journal.pone.0230880 (PMC7098606; doi:10.1371/journal.pone.0230880)
Supplement: S1 File — (DOCX) [file pone.0230880.s001.docx]

**S1 Table: English version of the questionnaires**

**Questionnaire ID No.__________**

**University of Gondar**

**College of medicine and health Sciences**

**Department of optometry**

A **questionnaire** prepared to collect data to assess Knowledge and its associated factors towards diabetes mellitus among community members of Gondar city, Ethiopia, 2019.

Structured questionnaire for determining knowledge and associated factors among adult non-diabetic community members about diabetes mellitus in Gondar city, Northwest Ethiopia.

Introduction

Good morning/afternoon, my name is -------------------------------- I am working in University of Gondar. I am a member of a research group working in University of Gondar. I am studying knowledge and associated factors among community members of Gondar city towards diabetes mellitus by asking questions. Your appropriate answers for all of our questions are important to know knowledge towards diabetes mellitus and its associated factors. Your answers will be confidential and keep in secret. If you decide that, you do not want to participate in the study now or at any time in the future; it is your right not to participate in the study. But we appreciate if you participate and will take 20 minutes for us to complete the questionnaire.

Thank you.

Do I have your permission to continue?

If yes thank you and continue --------------

If no, thank you and go to next study subject --------------

Data collector

Name ----------------------- signature ------------------------ date -------------------

Checked by supervisor

Name --------------------- signature---------------------- date-------------------

**Instruction: Please circle the number in front of the option the respondents choose & fill in the blank space that the respondent best describe in the right side of the table**.

| **Part-I: Respondent’s socio- demographic information** | | | |
| --- | --- | --- | --- |
| **S. No** | **Questions** | **Response options** | **Remarks** |
| 100 | Sex | **0** = Male  **1** = Female |  |
| 101 | Age | ________Years old |  |
| 102 | Marital status | **0** = Single  **1** = Married  **2** = Divorced/Separated  **3** = Widowed |  |
| 103 | Level of education | **0** = Unable to read and write  **1** = Able to read and write  **2** = Grade 1-8  **4** = Grade 9-12  **5**= College and above |  |
| 104 | Occupation | 0= House wife  1= Student  2 = Merchant  4 = Government/private employee  5= Daily laborer |  |
| 105 | Average family monthly income in ETB | _______________(in Ethiopian birr) |  |
| 106 | Have heard the disease “DM”? | 0 = Yes  1 = No |  |
| 107 | Exposure to health education about DM | 0 = Yes  1 = No |  |
| 108 | Have television/radio | 0 = Yes  1 = No |  |
| 109 | Family history of DM | 0 = Yes  1 = No  2 = Don’t know |  |
| 110 | If “Yes” for Q-106 your sources of information? | **0** = Medias  **1** = Health care workers  **2** = Friends/relatives  **3** = Others (teacher, religious leader…) |  |

| **Part-II: Knowledge questions related to DM** | | | | | |
| --- | --- | --- | --- | --- | --- |
| **S.No** | **Questions** | **Response options** | | | |
|  |  | **Yes =1** | **No**  **= 2** | **Do not know= 3** | **Remarks** |
|  | **What is/are DM** | **1** | **2** | **3** |  |
| 200 | DM is a condition of insufficient insulin production | **1** | **2** | **3** |  |
| 201 | DM is a condition of the body which not responding for insulin | **1** | **2** | **3** |  |
| 202 | DM is a condition of high level of sugar in the blood | **1** | **2** | **3** |  |
| 203 | DM is not curable | **1** | **2** | **3** |  |
| 204 | DM is diseases which affect any part of body | **1** | **2** | **3** |  |
|  | **What are the risk factors of DM** |  |  |  |  |
| 205 | Older age | **1** | **2** | **3** |  |
| 206 | Genetic or family history of diabetes mellitus | **1** | **2** | **3** |  |
| 207 | Being overweight /Obesity | **1** | **2** | **3** |  |
| 208 | Pregnancy | **1** | **2** | **3** |  |
| 209 | Sedentary life /Poor dietary habits | **1** | **2** | **3** |  |
| 210 | Not getting enough exercise can predispose to diabetes | **1** | **2** | **3** |  |
|  | **What are signs and symptoms of DM** |  |  |  |  |
| 211 | Frequent urination | **1** | **2** | **3** |  |
| 212 | Excessive thirst | **1** | **2** | **3** |  |
| 213 | Excessive hunger | **1** | **2** | **3** |  |
| 214 | Weight loss | **1** | **2** | **3** |  |
| 215 | High blood sugar | **1** | **2** | **3** |  |
| 216 | Blurred vision | **1** | **2** | **3** |  |
| 217 | Slow healing of cuts and wounds | **1** | **2** | **3** |  |
| 218 | Feeling of weakness | **1** | **2** | **3** |  |
|  | **Control and management DM** |  |  |  |  |
| 218 | Insulin injection is available for control and management of DM | **1** | **2** | **3** |  |
| 219 | Tablets & capsule are available for control and management of DM | **1** | **2** | **3** |  |
| 220 | Regular Exercise | **1** | **2** | **3** |  |
| 221 | Practices healthy diet | **1** | **2** | **3** |  |
| 222 | Medical eye checkup and care | **1** | **2** | **3** |  |
| 223 | Feet and toes medical checkup and care | **1** | **2** | **3** |  |
| 224 | Weight reduction | **1** | **2** | **3** |  |
|  | **Complications of DM** |  |  |  |  |
| 225 | Diabetes can cause eye problem or even blindness | **1** | **2** | **3** |  |
| 226 | Diabetes can cause kidney failure | **1** | **2** | **3** |  |
| 227 | Diabetes can cause heart failure | **1** | **2** | **3** |  |
| 228 | Diabetes can cause brain disease like Stroke | **1** | **2** | **3** |  |
| 229 | Diabetes can result in Amputation of limb | **1** | **2** | **3** |  |

**Thank you very much!!!!**

**S2 Table: Amharic version of the questionnaires**

**የጎንደር ዩኒቨርሲቲ ህክምናና ጤናሳይንስ ኮሌጅ የዓይን ህክምና ክፍል**

**መለያ ቁጥር -----------------**

**የመጠይቅ ቅፅ**

**ጤናይስጥልኝ -------------- እባላለሁ፡፡የጎንደር ዩነቨርስቲ ሠተራተኛና የዩነቭርስቲው የጥናት ቡድን አባል ነኝ፡፡ ስለስኳር በሽታ በጎንደር ከተማ የሚኖሩ ማህበረሰቦች ያላቸዉን እዉቀትና ተያያዥ ምክንያቶችን ቃለመጠይቅ በማድረግ እያጠናን እንገኛለን፡፡ይህ ጥናት እርስዎ በሚሰጡን መረጃ ላይ የተመሰረተ ስለሆነ ፍቃድዎ ከሆነ መረጃውን በመስጠት ትብብር እንዲያደርጉልን በትህትና እንጠይቃለን፡፡በጥናቱ ላይ መሳተፍ የማይፈልጉ ከሆነ አሁንም ሆነ በሂደት ወስጥ አለመስማማት ይችላሉ፡፡ሆኖም ግን ጥናቱ ከትንሽ ጊዜ መፍጀት ውጪ ምንም አይነት ጉዳት የማያመጣ ስለሆነ እንዲሳተፉ እናበረታተለን፡፡መረጃዎ ምስጢራዊነቱ የተጠበቀ ፣ለጥናቱ ብቻ የሚውልና ለሌላ ጉዳይ የማንጠቀምበት መሆኑን ልናረጋግጥልዎ እንወዳለን፡፡ቃለመጠይቁ 20 ደቂቃ የሚፈጅ ስለሆነ ፍቃደኝነትዎን በፊርማ እንዲያረጋግጡልንና ዉል እንዲወስዱልን በትህትና እየጠየቅን ወደ ቃለመጠይቁ እንሄዳለን፡፡**

**ለመሳተ ፍፈቃደኛ ከሆኑ ወደሚቀጥለው ገፅ ይለፉ.**

**ማንኛውም ሊያነሱ የሚፈልጉት ጥያቄ ካለዎት ተመራማሪውን በሚቀጥለው አድራሻ ማነጋገር ይችላሉ፡፡**

**ስም፡ ዐቢይ ማሩ**

**ስ. ቁ፡ 0918182413**

**መረጃውን የሰበሰበው**

**ስም ----------------------------------- ፊርማ -------------------------- ቀን -------------------**

**መረጃውን ያረጋገጠው**

**ስም----------------------------------- ፊርማ --------------------------- ቀን ------------------**

**መመሪያ:** እባክዎ መላሹ የሚሰጠዉን መልስ ከጥያቄዎች ፊት ለፊት ካሉት አማራጮቹ መካከል ያክብቡ ወይም መላሹ የሚገልፀዉን ሀሳብ በቀኝ በኩል ባለዉ ክፍት ቦታ ላይ ይፃፉ፡፡

| **ክፍል-አንድ: የጥናቱ ተሳታፊዎች ማህበራዊ ኩነታት መረጃዎች** | | | | | | | | |
| --- | --- | --- | --- | --- | --- | --- | --- | --- |
| **ተ.ቁ** | | **ጥያቄ** | **የመልስ አማራጮች** | | | | **ምርመራ** | |
| 100 | | ጾታ | **0** = ወንድ  **1** = ሴት | | | |  | |
| 101 | | ዕድሜ | ________ዓመት | | | |  | |
| 102 | | የጋብቻ ሁኔታ | **0** = ያላገባ  **1** = ያገባ  **2** = የፈታ/የፈታች/ለየብቻ የምኖሩ  **3** = የሞተባት/የሞተችበት | | | |  | |
| 103 | | የትምህርት ደረጃ | **0** = ማንበብናመፃፍ የማይችል  **1** = ማንበብናመፃፍ የሚችል  **2** = ክፍል 1-8  **4** = ክፍል 9-12  **5** = ኮሌጅ እና ከዚያ በላይ | | | |  | |
| 104 | | የስራ ሁኔታ | 0= የቤት እመቤት  1 = ተማሪ  2 = ነጋዴ  4 = የመንግስት/የግል ተቀጣሪ  5 = የቀን ሰራተኛ | | | |  | |
| 105 | | አማካይ የቤተሰብ ወርሃዊ ገቢ | _______________ብር ( ኢትዮጵያ ብር) | | | |  | |
| 106 | | ስለስኳር በሽታ ሰምተዉ ያዉቃሉ? | 0 = አዎ  1 = የለም | | | |  | |
| 107 | | ስለስኳር በሽታ የጤና ትምህርት አገኚተዉ ያዉቃሉ | 0 = አዎ  1 = የለም | | | |  | |
| 108 | | ቴሌቪዥን/ራዲዮ አለዎት | 0 = አዎ  1 = የለም | | | |  | |
| 109 | | በቤተሰብ ዉስጥ የስኳር በሽታ የተያዘ አለ | 0 = አዎ  1 = የለም  2 = አላዉቅም | | | |  | |
| 110 | | ለያቄ ቁጥር -106 መልስዎ “አዎ” ከሆነ መረጃዉን ያገኙት ከየት ነዉ? | **0** = መገናኛ ብዙሃን  **1** = የጤና ባለሞያ  **2** = ጓደኛ/ዘመድ  **3** = ሌላ (መምህር, የሀይማኖት አባት…) | | | |  | |
| **ክፍል-ሁለት: የስኳር በሽታ እዉቀትን የሚመለከቱ ጥያቄዎች** | | | | | | | | |
| **ተ.ቁ** | **ጥያቄዎች** | | | **የመልስ አማራጮች** | | | | |
|  |  |  |  | **አዎ =1** | **አይደለም**  **= 2** | **አላዉቅም= 3** | | **ምርመራ** |
|  | **የስኳር በሽታ ማለት ምን ማለት ነው ?** | | | **1** | **2** | **3** | |  |
| 200 | የስኳር በሽታ ማለት ከቆሽት የሚመነጨው ኢንሱሊን የተባለው ሆርሞን (ንጥረ ነገር) ጭራሽ መጥፋቱ/አለመመንጨቱ ወይም መጠኑ መቀነሱ | | | **1** | **2** | **3** | |  |
| 201 | የስኳር በሽታ ማለት ከቆሽት የሚመነጨው ኢንሱሊን የተባለው ሆርሞን (ንጥረ ነገር) የሚያከናውነው ሥራ ሲሰናከል የሚመጣ ነዉ። | | | **1** | **2** | **3** | |  |
| 202 | የስኳር በሽታ ማለት ስኳር ወይም ጉሉኮስ በደም ውስጥ ከመጠን በላይ ሆኖ ሲገኝ የሚከሰት ነው፡፡ | | | **1** | **2** | **3** | |  |
| 203 | የስኳር በሽታ መዳን የማይችል ነዉ፡፡ | | | **1** | **2** | **3** | |  |
| 204 | የስኳር በሽታ ሁሉንም የሰዉነት አካል የሚያጠቃ ነዉ | | | **1** | **2** | **3** | |  |
|  | **ለስኳር በሽታ መከሰት ምክንያት ሊሆኑ የሚችሉ ጠንቆች አሉ?** | | |  |  |  | |  |
| 205 | የዕድሜ መጨመር (በእድሜ መግፋት) | | | **1** | **2** | **3** | |  |
| 206 | ዘር ወይም በቤተሰብ ውስጥ ሕመሙ ቀደም ብሎ መታየቱ | | | **1** | **2** | **3** | |  |
| 207 | ዉፍረት(ውፋሬ) ወይም ክብደት ከሚገባ በላይ መጨመር | | | **1** | **2** | **3** | |  |
| 208 | በርግዝና ወቅት በስኳር በሽታ መታመም | | | **1** | **2** | **3** | |  |
| 209 | የልተስተካከል የኑሮ ሁኔታ/የአመጋገብ ሁኔታ | | | **1** | **2** | **3** | |  |
| 210 | በቂ እንቅስቃሴ አለማግኘት | | | **1** | **2** | **3** | |  |
|  | **የስኳር በሽታ ምልክቶች ምንድን ናቸው?** | | |  |  |  | |  |
| 211 | አዘውትሮ /ቶሎ ቶሎ መሽናት | | | **1** | **2** | **3** | |  |
| 212 | በብዛት ውሃ መጠጣት | | | **1** | **2** | **3** | |  |
| 213 | በብዛት መራብ | | | **1** | **2** | **3** | |  |
| 214 | በብዛት ክብደት መቀነስ (መክሳት) | | | **1** | **2** | **3** | |  |
| 215 | በደም ዉስጥ ያለዉ ስኳር መጠን በብዛት መጨመር | | | **1** | **2** | **3** | |  |
| 216 | የዓይን ግርዶሽ | | | **1** | **2** | **3** | |  |
| 217 | በሰዉነት ላይ ያሉ ቁስሎች ቶሎ አለመዳን/ከቁስል በቶሎ ለመዳን አለመቻል | | | **1** | **2** | **3** | |  |
| 218 | የድካም ስሜት | | | **1** | **2** | **3** | |  |
|  | **የስኳር በሽታንና ጠንቆቹን እንዴት መከላከል ወይም እንዳይባባሱ ማድረግ ይቻላል?** | | |  |  |  | |  |
| 218 | በመርፌ መልክ በሚወሰዱ የተለያዩ የኢንሱሊን ዝግጅቶች የስኳር በሽታን መቆጣጠር ይቻላል | | | **1** | **2** | **3** | |  |
| 219 | በአፍ በሚወሰዱ በክኒን እና በጥቅል መልክ ባሉ መድሀኒቶች የስኳር በሽታን መቆጣጠር ይቻላል | | | **1** | **2** | **3** | |  |
| 220 | አዘውትሮ የሰውነት እንቅስቃሴ (ስፖርት) መሥራት። ለምሳሌ ያህል በየቀኑ ግማሽ ስዓት በመውሰድ በሳምንት ውስጥ ለአምስት ቀናት ያህል መሮጥ | | | **1** | **2** | **3** | |  |
| 221 | ጤነኛ የሆነ ምግብ መመገብ(ይህ ምግብ አትክልትና ፍራፍሬ በብዛት የያዘ ሆኖ) | | | **1** | **2** | **3** | |  |
| 222 | የዓይንን ጤንነት በሕክምና አዋቂ መመርመርና መንከባከብ | | | **1** | **2** | **3** | |  |
| 223 | የእግርን ጤንነት በሕክምና አዋቂ መመርመርና መንከባከብ | | | **1** | **2** | **3** | |  |
| 224 | ተገቢ በሆነ የሰውነት ክብደት ወይም ውፋሬ ላይ መገኘት | | | **1** | **2** | **3** | |  |
|  | **የስኳር በሽታ ጥንቃቄ ሳይደረግለት እንዲቀጥል ከተደረገ ምን ችግሮችን ያስከትላል?** | | |  |  |  | |  |
| 225 | የአይን በሽታን ብሎም መታወርን ማስከተል | | | **1** | **2** | **3** | |  |
| 226 | የኩላሊት በሽታን ማምጣት ወይም ማባባሰ | | | **1** | **2** | **3** | |  |
| 227 | የልብ በሽታ መከሰት | | | **1** | **2** | **3** | |  |
| 228 | በአንጎል ውስጥ ደም የመፍሰስ ወይም የመርጋት ችግር መፍጠር | | | **1** | **2** | **3** | |  |
| 229 | ለእግር መታመምና መቆረጥ ምክንያት መሆን | | | **1** | **2** | **3** | |  |

**ጨርሰናል! በጣም አመሰግናልሁ!!!!**
